# Assessment of financial protection mechanisms for tuberculosis services in seven priority countries of the Western Pacific Region: a cross-sectional mixed-methods study

**DOI:** 10.1186/s41182-026-00979-7

**Published:** 2026-08-06

**Authors:** Kyung Hyun Oh, Jeungeun Seo, Yerin Yu, Fukushi Morishita, Alia Cynthia Luz, Huong Thi Giang Tran, Rajendra Prasad Hubraj Yadav, Martin P. Grobusch, Hongjo Choi

**Affiliations:** 1 World Health Organization Regional Office for the Western Pacific, Manila, Philippines; 2 Department of Infectious Disease, Division of Internal Medicine, Amsterdam University Medical Center, University of Amsterdam, Amsterdam, Netherlands; 3 Division of Health Policy and Management, Korea University College of Health Science, Seoul, Republic of Korea

## Abstract

**Background:**

Tuberculosis (TB) remains a major public health and socioeconomic challenge in the Western Pacific Region. Despite the expansion of universal health coverage (UHC), TB-affected households continue to face financial hardship. This study aimed to assess and compare financial protection mechanisms for TB services across the continuum of care in the region.

**Methods:**

We conducted a cross-sectional mixed-methods study in seven TB priority countries in the Western Pacific Region: Cambodia, China, Lao People’s Democratic Republic, Mongolia, Papua New Guinea, the Philippines, and Viet Nam. Data were collected between January and March 2025 through a structured online questionnaire and semi-structured interviews with 21 key informants. Findings were analysed using a standardised health financing framework covering revenue raising, pooling, purchasing, and service coverage. Secondary data from the WHO Global TB finance database were used to assess funding composition and donor dependence.

**Results:**

Countries were mapped into three financing typologies: national health service-type systems, government-subsidised mixed systems, and insurance-based pooled systems. Total TB funding in 2023 varied widely, from US$3.7 million in the Lao People’s Democratic Republic to US$1.08 billion in China. External donor dependence was high in the Lao People’s Democratic Republic, Viet Nam, Cambodia, and Papua New Guinea, but minimal in China. Coverage of TB services was strongest for drug-susceptible TB treatment and weakest for radiology, hospitalisation, and services subject to social health insurance co-payments. TB preventive treatment and drug-resistant TB services were frequently sustained through donor financing. Interviews identified implementation barriers including pre-diagnostic user fees, reimbursement delays, fragmented purchasing, and incentives for unnecessary hospitalisation.

**Conclusions:**

Expansion of UHC has not consistently translated into comprehensive financial protection for TB patients. While many TB services are nominally covered, patients remain exposed to costs through pre-diagnostic fees, co-payments, reimbursement limits, and donor-dependent service components. Strengthening financial protection requires integrating TB services into pooled domestic financing, removing avoidable cost-sharing for essential TB services, and aligning purchasing mechanisms with patient-centred TB care pathways.

**Supplementary Information:**

The online version contains supplementary material available at https://doi.org/10.1186/s41182-026-00979-7.

## Background

Tuberculosis (TB) remains a major public health and socioeconomic challenge in the Western Pacific Region, accounting for 27% of the global TB burden in 2024 [1]. Despite progress in expanding access to diagnosis and treatment, TB continues to expose affected individuals and households to financial hardship. The prolonged duration of treatment, frequent health facility visits, and underlying socioeconomic vulnerability contribute to the risk of catastrophic costs, even in settings where TB services are nominally provided free of charge [2].

Financial protection is a core objective of universal health coverage (UHC), defined as ensuring access to needed health services without incurring financial hardship [3]. Health financing arrangements, encompassing how resources are raised, pooled, and allocated to purchase services, are central to achieving this objective [4]. Recognising this, the WHO End TB Strategy places financial protection at the centre of the global TB response and sets a target of zero TB-affected households facing catastrophic costs by 2030 [5]. This objective is reinforced in the Western Pacific regional framework to end TB: 2021–2030, which emphasises the integration of TB services within broader UHC and social protection systems [6, 7].

Over the past two decades, countries in the Western Pacific Region have undertaken major health financing reforms aimed at moving toward UHC. These reforms include the expansion of tax-funded public services, the introduction or scale-up of social health insurance (SHI) schemes, and the increased use of targeted government subsidies [8, 9]. While they aim to reduce reliance on out-of-pocket (OOP) payments and to strengthen financial risk protection, evidence from TB patient cost surveys indicates that financial hardship remains widespread across diverse health system contexts [2]. Nine surveys conducted to date in the region reveal that the proportion of TB-affected households facing catastrophic costs ranges widely from 34% in Cambodia to 92% in the Solomon Islands [1, 2]. Although non-medical costs and income loss are major contributors to catastrophic costs [10], the design and implementation of financing mechanisms for TB medical services under UHC represent a necessary foundation for comprehensive financial protection.

Health financing architectures in the region are highly heterogeneous. For public service provision, some countries rely predominantly on national health service-type systems, others operate mixed government-subsidised models with contributory or non-contributory arrangements, while several have established contributory SHI systems as the backbone of health financing [8, 9]. Despite progress, insufficient government health spending and fragmented funding streams continue to drive systemic inefficiencies. These insufficiencies are often exacerbated by parallel donor-funded vertical programmes [11]. Furthermore, primary healthcare, essential for equitable TB service delivery, remains chronically underfunded [11]. These structural flaws directly shape TB benefit packages, patient cost-sharing, and donor reliance. Consequently, it is essential to understand how they affect financial protection across the TB care continuum.

Existing literature on TB financing in the Western Pacific Region has established the magnitude of financial hardship for patients [12], tracked macro-level trends in national TB programme financing [13], and outlined the policy roadmap for integrating TB into UHC [14]. However, there remains a lack of systematic evidence comparing how specific national financing architectures purchase and cover TB services across the full continuum of care. Furthermore, despite historical debates over disease-specific ‘vertical’ funding versus health system integration, it remains unclear how these macro-level choices translate into patient-level financial barriers today [15]. This gap is particularly salient in the region, where countries are at different stages of UHC reform and face varying levels of dependence on external assistance.

This study therefore aims to assess and compare financial protection mechanisms for TB services across the full continuum of care in the Western Pacific Region.

## Methods

### Study design and setting

We conducted a cross-sectional mixed-methods study to assess financial protection mechanisms for TB services in seven priority countries of the Western Pacific Region. Based on 2023 WHO estimates [16], inclusion criteria were: (1) an absolute burden of ≥ 40,000 TB cases, or (2) ≥ 10,000 cases with an incidence of ≥ 100 per 100,000 population. Five countries met the first criterion: China (~ 741,000 cases), the Philippines (~ 739,000), Viet Nam (~ 182,000), Cambodia (~ 58,000), and Papua New Guinea (~ 45,000). Two met the second criterion: Mongolia (448/100,000; ~ 15,000 cases) and Lao People’s Democratic Republic (Lao PDR) (133/100,000; ~ 10,000 cases). Countries lacking WHO Western Pacific Region membership at the time of the study (e.g., Indonesia) or failing to meet these burden thresholds were excluded.

### Data collection

Data were collected in two sequential phases from focal points representing national TB programmes, health financing or SHI agencies, and WHO country offices, comprising a total of 21 key informants (three representatives from each of the seven target countries).

First, a structured online questionnaire (Supplementary 1) was administered between January and February 2025. The questionnaire collected information on financial protection arrangements for TB services across all relevant health financing schemes operating within each country. For each applicable scheme, respondents reported on revenue-raising sources (including government funding, contributory mechanisms where applicable, and external donor financing), purchasing and reimbursement arrangements, and coverage of TB services along the continuum of care. Service domains included TB prevention, diagnosis, treatment, hospitalisation, and selected patient support services delivered through the health system. Respondents also reported the extent of service coverage and the lowest level of health facility at which services were available.

Second, semi-structured follow-up interviews with the survey respondents were conducted virtually between February and March 2025 using a standardised interview guide (Supplementary 2). The interviews aimed to clarify survey responses and explore implementation experiences in greater depth. Interview topics included interactions between TB programmes and broader health financing arrangements, interpretation of reported coverage and cost-sharing practices, reliance on external donor financing, and perceived gaps in financial protection for TB services. Interviews were audio-recorded with consent and transcribed verbatim.

To ensure robustness, self-reported findings were verified through a targeted review of relevant literature, encompassing national TB strategic plans, health financing policy documents, and peer-reviewed publications.

To contextualise the primary structural data, we extracted secondary data on national funding for TB prevention, diagnosis, and treatment services from the WHO Global TB finance database for the year 2023 [16]. For each of the seven study countries, we compiled the annual data on total funding for these services, disaggregated by source: domestic funding (including both direct budgets reported by national TB programmes and the imputed financial costs of inpatient and outpatient care) and external funding (specifically including the Global Fund, USAID, and other grants).

### Data analysis

**Fig. 1:**
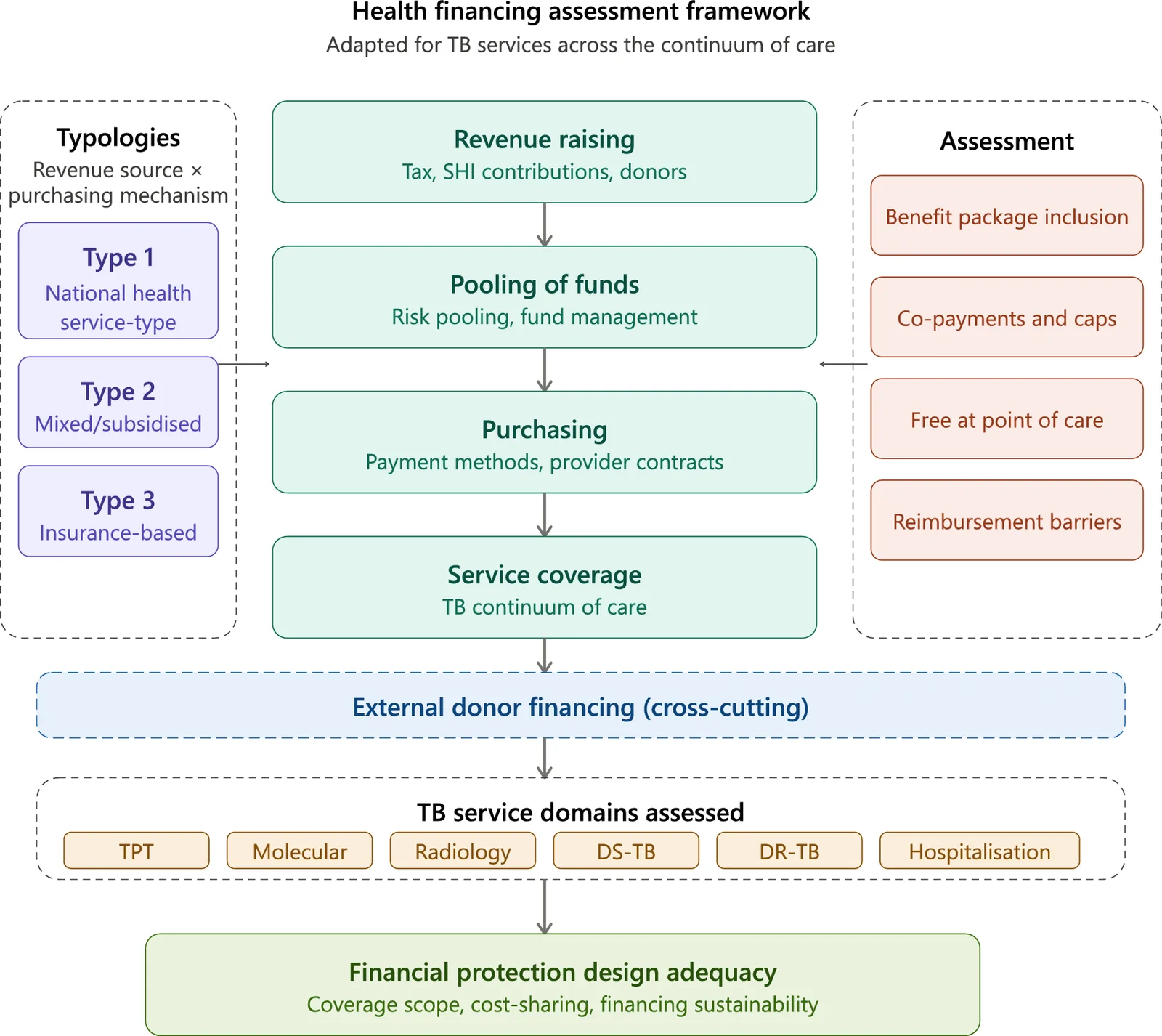
Health financing assessment framework. Each TB service domain represents a key node in the continuum of care. TPT denotes preventive treatment for individuals at risk of progression to active disease. Molecular diagnostics include WHO-recommended rapid tests (e.g., Xpert MTB/RIF). Radiology refers to chest X-ray for screening or diagnostic workup. DS-TB and DR-TB management and care encompass drug regimens, clinical monitoring, and ancillary investigations during the treatment course. Hospitalisation refers to inpatient admission fees and associated charges, whether short-stay or long-term. SHI: social health insurance; TPT: tuberculosis preventive treatment; DS-TB: drug-susceptible tuberculosis; DR-TB: drug-resistant tuberculosis

Data were analysed using a standardised health financing framework covering revenue raising, pooling, purchasing, and service coverage (Fig. 1). For the comparative analysis, we deductively defined three a priori typologies based on established global health financing literature [4, 17]: national health service-type systems (Type 1), government-subsidised mixed systems (Type 2), and insurance-based pooled systems (Type 3). The typologies serve as analytical categories for organising cross-country comparison; they are not fixed or exhaustive classifications. Within-type variation in coverage outcomes reflects country-specific benefit design decisions, not a failure of the financing architecture model. This allowed us to systematically map the seven countries against theoretical models based on their empirical financing arrangements. External donor financing was treated as a cross-cutting feature interacting with all three typologies. The extracted secondary funding data were subsequently used to quantify the degree of donor dependence across the seven countries and assess the structural sustainability of current coverage arrangements.

Potential financial protection was evaluated based on system design—specifically the inclusion of TB services in benefit packages, point-of-care fees, and co-payments—rather than observed patient expenditure. The analytical focus is deliberately placed on the upstream financing architecture for medical services, as identifying which structural design features create cost exposure is a prerequisite for targeted policy reform.

Non-medical costs and income loss were excluded from this analytical scope. While these costs are recognised as major contributors to catastrophic expenditure among TB-affected households [10], they fall outside the direct assessment of medical financing mechanisms. These broader dimensions of financial hardship are comprehensively evaluated in our complementary regional analysis [18].

To maintain a rigorous mixed-methods design, we separated data streams. Quantitative and structural data from questionnaires and secondary databases were used exclusively to map policy design and financing typologies, while qualitative interviews were analysed thematically to identify de facto implementation barriers and perverse incentives. Findings from all data sources were subsequently triangulated to assess convergence, clarify policy–practice gaps, and generate a comprehensive regional comparison.

### Ethical considerations

The study protocol was reviewed by the WHO Regional Office for the Western Pacific Ethics Review Committee and deemed exempt from full ethical review (Protocol ID: 2025.1.ICP.1.ETB), as it involved interviews with public officials in their official capacity on topics in the public domain. Participation was voluntary, informed consent was obtained from all interviewees, and no personally identifiable data were collected.

## Results

### Overview of financing architecture

Based on revenue sources and purchasing arrangements, countries were mapped into the three a priori financing typologies (Box): (1) national health service-type system (Papua New Guinea); (2) government-subsidised mixed system (Cambodia and Lao PDR); and (3) predominantly insurance-based pooled system (China, Mongolia, the Philippines, and Viet Nam).

Box. Typology of health financing architectures for tuberculosis services in seven priority countries in the Western Pacific Region **Type 1 National health service-type system**

TB services are financed predominantly through general government revenues pooled within the public budget and delivered through publicly managed providers. Purchasing is largely passive, typically through line-item budgeting. Formal health insurance mechanisms either do not exist or play a negligible role in pooling and purchasing TB services. External donor financing frequently supplements domestic allocations.

- Papua New Guinea

**Type 2. Government-subsidised mixed system**

TB services are financed primarily through government budget allocations and external donor support, even where health insurance agencies exist within the broader health system. In these settings, insurance schemes may pool resources and reimburse selected services; however, TB diagnostics, drugs, and core programme components remain largely funded through government subsidies and vertical allocations. Purchasing tends to remain input-oriented or programme-based, with partial insurance integration.

- Cambodia, Lao People’s Democratic Republic

**Type 3. Predominantly insurance-based pooled system**

TB services are purchased primarily through social health insurance agencies that function as the main pooling and purchasing platforms within the health system. Government budget transfers may be channelled through these insurance pools, and external financing may continue to support specific components (e.g., drug-resistant TB). In the past few decades, pooling multiple revenue sources under insurance schemes in WPR countries has allowed more strategic purchasing mechanisms, such as capitation, case-based payment, or DRG systems, to play a central role in financing TB service delivery.

- China, Mongolia, the Philippines, Viet Nam

TB = tuberculosis; DRG = diagnosis-related group.

### Funding for TB prevention, diagnostic and treatment services by source

Total funding for TB prevention, diagnostic, and treatment services in 2023 ranged from US$ 3.7 million in Lao PDR to US$ 1081.3 million in China (Table 1). The proportional composition of these funding sources varied significantly across the seven countries. Four countries relied heavily on external donors, primarily the Global Fund: external resources accounted for 70.2% of the total TB budget in Lao PDR, 62.9% in Viet Nam, 60.5% in Cambodia, and 60.1% in Papua New Guinea. The Philippines exhibited moderate dependence, with 42.0% of its budget financed externally. Conversely, two countries funded their programmes predominantly through domestic sources; external funding comprised 20.6% of the budget in Mongolia and only 0.1% in China.

**Table 1:** Funding for tuberculosis prevention, diagnostic and treatment services by source, seven priority countries in the Western Pacific Region, 2023

Country	Total (US$ thousands)	Domestic funding (US$ thousands)			External funding by source (US$ thousands)				External share (%)
		NTP-reported	IP/OP imputed	Subtotal	Global Fund	USAID	Other grants	Subtotal	
Cambodia*	20,384	6500	1554	8054	7000	4000	1330	12,330	***60.5***
China	1,081,340	763,442	316,960	1,080,402	0	0	938	938	*0.1*
Lao PDR	3748	577	540	1117	2631	0	0	2631	***70.2***
Mongolia	14,781	10,961	781	11,742	3032	0	7	3039	**20.6**
Papua New Guinea*	15,590	3947	2280	6227	8,278	0	1085	9363	***60.1***
Philippines	176,083	82,534	19,645	102,179	51,904	22,000	0	73,904	**42.0**
Viet Nam	67,892	9048	16,152	25,200	40,564	879	1249	42,692	***62.9***

Source: WHO global TB database. Geneva: World Health Organization; data extracted 30 April 2025. Available from: https://www.who.int/teams/global-programme-on-tuberculosis-and-lung-health/data

Methodology: Following the WHO definition, domestic funding includes (i) amounts reported by national TB programmes (NTPs) plus (ii) the estimated financial costs of inpatient and outpatient (IP/OP) care required during TB treatment. IP/OP costs were imputed as the product of treatment volumes and the corresponding cost-per-patient values for 2023; for Cambodia and Papua New Guinea, which did not submit expenditure data for 2023, budget-side cost-per-patient values were used. External funding includes Global Fund grants, USAID bilateral disbursements, and other international grants as reported by NTPs. External share = (external subtotal/total) × 100%

All figures expressed in US$ thousands. Countries marked with * (Cambodia, Papua New Guinea) did not submit expenditure data for 2023; values for these countries reflect committed funding on the external side and budget-derived cost-per-patient on the IP/OP imputation side. Within-country totals may not exactly match the sum of individual sources due to rounding

Italics: external share ≤ 20% (low vulnerability); bold: 21–50% (moderate); bold italics: > 50% (high vulnerability)

### Purchasers, providers, and payment mechanisms

Purchasing arrangements, providers, and payment mechanisms for TB services vary across the seven countries (Table 2). Ministries of health and SHI agencies serve as purchasers in five countries each. Cambodia and Lao PDR additionally rely on international donors as purchasers. Service provision predominantly occurs in public general or referral hospitals (six countries) and primary care facilities (five countries). China and the Philippines also utilise designated TB hospitals or private clinics.

**Table 2:** Purchasers, providers, and payment mechanisms for tuberculosis services in seven priority countries of the Western Pacific Region

Category	Sub-category	Countries (n = 7)	List of countries
Purchasers	Ministry of Health/National TB Programme	5	Cambodia, Lao PDR, Mongolia, Papua New Guinea, the Philippines
	Social health insurance agency (NHI, VSS, PhilHealth)	5	China, Lao PDR, Mongolia, the Philippines, Viet Nam
	Global Fund/international donors	2	Cambodia, Lao PDR
Providers	Primary care facilities (health centres)	5	Cambodia, Lao PDR, Mongolia, Papua New Guinea, Viet Nam
	Public general or referral hospitals	6	Cambodia, China, Lao PDR, Mongolia, Papua New Guinea, Viet Nam
	Designated TB hospitals or private clinics	2	China, the Philippines
Payment mechanisms	Line-item budgeting (input-based)	3	Cambodia, Lao PDR, Papua New Guinea
	Case-based payment, DRGs, or service packages	5	Cambodia, China, Mongolia, the Philippines, Viet Nam
	Capitation	2	Mongolia, Viet Nam
	Fee-for-service	2	China, Viet Nam

Categories are non-mutually exclusive; countries may use multiple purchasers, providers, and payment mechanisms in parallel for different components of TB services. Rows are ordered within each category by the number of countries reporting use, descending. NHI: National Health Insurance (Lao PDR); VSS: Vietnam Social Security; PhilHealth: Philippine Health Insurance Corporation; DRG: diagnosis-related group

Regarding payment mechanisms, traditional input-based line-item budgeting is used in three countries (Cambodia, Lao PDR, and Papua New Guinea). Conversely, strategic purchasing models such as case-based payments, diagnosis-related groups (DRGs), or service packages are utilised in five countries (Cambodia, China, Mongolia, the Philippines, and Viet Nam). Notable examples include the PhilHealth ‘TB-DOTS Package’ in the Philippines, DRG and diagnosis-intervention packet (DIP) reforms in China, and case-based Health Equity Funds (HEFs) in Cambodia. Capitation (e.g., for primary care in Mongolia and Viet Nam) and fee-for-service models (China, Viet Nam) are each employed in two countries.

### Coverage of TB services along the continuum of care

Financial protection for TB services varies significantly across the care continuum depending on the service domain and national funding architecture (Table 3). TB preventive treatment (TPT) is fully covered in six countries. This is split evenly between donor-funded mechanisms (Cambodia, Lao PDR, Papua New Guinea) and domestic government/SHI schemes (Mongolia, the Philippines, Viet Nam). In contrast, China currently excludes TPT from standard benefit packages, leaving patients fully exposed to OOP costs. Coverage for diagnostics diverges by modality. Molecular diagnostics are fully covered in five countries but require partial SHI co-payments in China and Viet Nam. Radiology (chest X-ray) presents greater financial vulnerability: only three countries (Mongolia, Papua New Guinea, Viet Nam) offer full statutory coverage. The remaining four countries (Cambodia, China, Lao PDR, the Philippines) provide only partial coverage, requiring patients to navigate OOP payments or co-payments unless they can access specific donor vouchers.

**Table 3:** Coverage of tuberculosis services across the continuum of care in seven priority countries of the Western Pacific Region

Service domain	Coverage (funding type)	Countries (n = 7)	List of countries
TPT	*Full (donor-funded)*	3	Cambodia, Lao PDR, Papua New Guinea
	*Full (government/SHI)*	3	Mongolia, the Philippines, Viet Nam
	***None (OOP)***	1	China
Molecular diagnostics	*Full (donor/government/SHI)*	5	Cambodia, Lao PDR, Mongolia, Papua New Guinea, the Philippines
	**Partial (SHI with co-payment)**	2	China, Viet Nam
Radiology (chest X-ray)	*Full (government/SHI)*	3	Mongolia, Papua New Guinea, Viet Nam
	**Partial (mixed funding with OOP or co-payment)**	4	Cambodia, China, Lao PDR, the Philippines
DS-TB treatment	*Full (government/donor/SHI)*	5	Cambodia, Lao PDR, Mongolia, Papua New Guinea, the Philippines
	**Partial (SHI with co-payment)**	2	China, Viet Nam
DR-TB treatment	*Full (donor-funded)*	4	Cambodia, Lao PDR, Mongolia, Papua New Guinea
	*Full (government/SHI)*	1	the Philippines
	**Partial (SHI with co-payment)**	2	China, Viet Nam
Hospitalisation	*Full (government/SHI)*	4	Cambodia, Lao PDR, Mongolia, Papua New Guinea
	**Partial (SHI with co-payment)**	3	China, the Philippines, Viet Nam

Coverage classification reflects the dominant funding arrangement reported by national focal points for each service domain. Full: service available without patient cost at the point of care; Partial: service available with co-payments, deductibles, or scheme-specific reimbursement limits; None: service not covered, full out-of-pocket payment required

Italics: Full coverage; bold: Partial coverage; bold italics: None

TPT: tuberculosis preventive treatment; SHI: social health insurance; OOP: out-of-pocket; DS-TB: drug-susceptible tuberculosis; DR-TB: drug-resistant tuberculosis

Five countries (Cambodia, Lao PDR, Mongolia, Papua New Guinea, and the Philippines) provide full coverage for drug-susceptible TB (DS-TB) through mixed government, donor, or SHI funds. Conversely, China and Viet Nam offer only partial coverage, as DS-TB treatment remains subject to mandatory SHI co-payments. For drug-resistant TB (DR-TB), four countries (Cambodia, Lao PDR, Mongolia, Papua New Guinea) rely entirely on full donor funding. The Philippines provides full government/SHI coverage for DR-TB, whereas China and Viet Nam again utilise partial SHI coverage with co-payments. Finally, inpatient hospitalisation is fully covered by government or SHI funds in four countries (Cambodia, Lao PDR, Mongolia, Papua New Guinea), though standard packages may still exclude ancillary medical supplies. The remaining three countries (China, the Philippines, Viet Nam) offer partial coverage due to deductibles or mandatory SHI co-payments, such as Viet Nam’s statutory 20% co-payment rate.

### Qualitative implementation barriers

Qualitative interviews revealed distinct implementation challenges depending on the financing architecture. In Cambodia, Lao PDR, and Papua New Guinea, the primary barrier is the ‘diagnostic gap’. Respondents described a pathway where the ‘free’ TB system effectively begins only after a confirmed diagnosis. Consequently, patients bear the entire cost of the initial investigative consultation, chest X-rays, and nonspecific antibiotic trials. Furthermore, informants highlighted that because TPT requires pre-initiation clinical workups (such as chest X-rays and liver function tests) that fall outside the free TB programme mandate, patients frequently incur user fees before TPT can even begin. In Papua New Guinea, the rigidity of vertical funding was cited as a major inefficiency; interviewees noted instances where free TB drugs were available at the provincial level but could not be transported to district facilities due to a lack of flexible operational funds for fuel.

In the Philippines and Viet Nam, the challenges are largely administrative and financial. Respondents reported that ‘reimbursement lags’, delays in payments from the insurance agency to health facilities, compromise facility cash flow. To mitigate this, providers reportedly resort to demanding upfront cash deposits from patients or ‘balance billing’ beyond allowed rates. Furthermore, integrating TB into SHI has created a ‘missing middle’ for ancillary operational costs. In Viet Nam, as donor support declines, the domestic insurance fund is legally prohibited from covering certain non-clinical expenditures such as sample transportation, effectively shifting these operational costs back to patients as de facto user fees. Interviewees also noted that mandatory cost-sharing, such as Viet Nam’s 20% inpatient co-payment and hospital fees for chest X-rays, creates a significant OOP burden for patients requiring long hospital stays.

In Mongolia, qualitative findings highlighted barriers stemming from strategic purchasing design, specifically regarding provider payment mechanisms. While full inpatient coverage was nominally designed to incentivise treatment adherence, key informants indicated that the SHI system’s reliance on DRGs for inpatient reimbursement has inadvertently created perverse financial incentives. To maximise facility revenue, providers reportedly encourage unnecessary hospitalisations for clinically stable TB patients. This practice not only strains the overall insurance fund, but also shifts unnecessary co-payments and indirect inpatient costs onto patients, fundamentally undermining the cost-efficiency and financial protection of the outpatient care model.

In China, implementation barriers centre on the administrative complexity of its decentralised SHI system. Key informants noted that fragmented insurance pools at the municipal level create significant reimbursement hurdles, particularly for internal migrants (*hukou*). Furthermore, complex procedures and strict caps for outpatient benefits often force patients to either absorb OOP costs or seek unnecessary hospitalisations to access more generous inpatient reimbursement rates, thereby undermining the intended financial protection.

## Discussion

This study provides a comparative typology of TB financing architectures across seven priority countries in the Western Pacific Region. It reveals a critical discrepancy between the maturing general health systems in these countries and the persistent fragility of financial protection for TB patients. While all seven nations have largely committed to UHC, our findings demonstrate that nominal coverage, or the legal entitlement to free care, does not equate to effective financial protection. The classification of countries into national health service-type (Type 1), mixed/subsidised (Type 2), and insurance-based (Type 3) systems highlights that while significant progress has been made in eliminating direct fees for TB drugs, the nature of financial barriers has evolved, shifting towards indirect access costs and systemic co-payments [8].

Empirical outcomes highlight the persistent financial challenges faced by TB patients across the region. National TB patient cost surveys document high catastrophic cost prevalence, including 34% in Cambodia, 34% in Papua New Guinea, 42% in the Philippines, 63% in Viet Nam, 63% in Lao PDR and 70% in Mongolia [1, 2]. While these surveys indicate that non-medical costs and income loss are often the predominant drivers of overall financial hardship [10], the structural gaps in medical financing architectures identified in our study likely contribute to this broader burden. Our typology explores how these specific medical financing vulnerabilities manifest across different system designs.

In Type 1 and Type 2 settings, specifically Cambodia, Lao PDR, and Papua New Guinea, the primary driver of financial vulnerability is the diagnostic gap. Although treatment costs are fully subsidised by donors, the pre-diagnostic pathway remains subject to user charges. Our findings corroborate recent national patient cost surveys indicating that a significant proportion of catastrophic costs are incurred before a TB diagnosis is confirmed [19, 20]. Because the free TB programme is vertically siloed, patients navigating the general health system with nonspecific symptoms are often subjected to outpatient user fees, chest X-ray charges, and costs associated with empirical antibiotic treatment [21]. This structural disconnect effectively imposes an entry fee to nominally free TB services.

Furthermore, the rigidity of line-item budgeting in Papua New Guinea mirrors findings from sub-Saharan Africa where inflexible public financial management systems result in liquidity stockouts, such as a lack of fuel, rendering physically available commodities inaccessible to patients [22]. These findings empirically validate long-standing concerns regarding the rigidity of vertical programming. As noted in case studies from Lao PDR and Papua New Guinea over a decade ago, while the Global Fund investments successfully scaled up treatment, they often reinforced parallel systems for procurement and reporting, ultimately leaving the broader primary care diagnostic capacity under-resourced [23, 24].

For Type 3 countries, including China, Mongolia, the Philippines, and Viet Nam, the transition to SHI presents what we term a UHC paradox. In theory, SHI deepens financial protection by pooling resources. In practice, standard insurance benefit designs apply cost-sharing mechanisms, such as co-payments and deductibles, to manage moral hazard and ensure fund solvency. The paradox arises when this insurance logic is applied to TB. Because TB requires long-term, high-frequency clinical management, cost-sharing rules calibrated for episodic acute care aggregate into a severe burden, acting as a regressive tax on the poor. Our empirical mapping shows that despite advanced institutional architectures, Type 3 countries inadvertently institutionalise financial barriers through systemic reliance on cost-sharing.

This structural exposure is perfectly illustrated by the empirical outcomes in China, which has transitioned to predominantly domestic funding. In this setting, direct medical costs remain a primary driver of catastrophic expenditure, accounting for 69% of total patient costs [25], directly reflecting the structural exposure identified in our Type 3 framework. While these countries have achieved administrative integration of TB into general health insurance (thereby moving away from vertical silos), they have not yet achieved the functional adaptation required to protect vulnerable patients [26, 27]. The shift from vertical to horizontal financing has, in some contexts, exposed TB patients to the co-payments inherent in general insurance schemes, confirming that integration without targeted financial protection mechanisms can regressively impact access [26].

Moreover, purchasing mechanisms can introduce perverse incentives if not accompanied by appropriate regulatory oversight. Mongolia’s use of DRGs for inpatient care has incentivised unnecessary hospitalisations [28], a phenomenon well-documented during health system transitions in Eastern Europe [29]. Conversely, the case-based payment model in the Philippines creates a coverage gap where complications or adverse events falling outside the standard package become the patient’s financial liability. This underscores the importance of Kutzin’s framework for strategic purchasing, which argues that payment methods must be linked to clinically appropriate pathways rather than just administrative convenience [17].

The integration of macro-level funding data quantifies a critical distinction between nominal service coverage and systemic sustainability. While our service-level assessment identified broadly comparable coverage across Type 1, Type 2, and some Type 3 countries (Table 3), the underlying financing sources differ fundamentally. In Cambodia, Lao PDR, and Papua New Guinea, 60–70% of TB financing originates from external donors. Consequently, services classified as “Full” coverage in these settings are structurally fragile. This funding analysis reveals a severe vulnerability within the typology: it is insufficient to achieve nominal coverage if the financing source that enables it is time-limited.

Crucially, this fragility extends into the Type 3 insurance-based systems. While China’s near-complete domestic financing (99.9%) demonstrates that an insurance-based architecture can sustain comprehensive TB budgets without external support, other Type 3 countries remain highly vulnerable. Notably, Viet Nam and the Philippines still rely on external sources for 62.9% and 42.0% of their respective TB budgets. As these countries face narrowing Global Fund eligibility, this substantial external funding share will need to be rapidly absorbed by domestic purchaser–provider agreements and pooled financing systems rather than maintaining parallel donor-funded supply chains [30]. The implementation barriers identified, specifically the reimbursement lags in insurance systems, suggest that public financial management systems must be strengthened to handle the cash-flow requirements of high-volume TB providers before donor funds are fully phased out.

While this structural analysis primarily highlights system vulnerabilities, the typologies also reveal successful policy innovations that serve as positive regional benchmarks. Regarding financial sustainability, China’s achievement of near-100% domestic financing demonstrates that transitioning to a fully self-reliant architecture for TB is operationally attainable. In terms of benefit design, Mongolia’s statutory exemption of co-payments for inpatient TB care represents a structural best practice, correctly recognising TB as a public health priority that requires maximum subsidy to protect patients. Furthermore, regarding the protection of vulnerable populations, Cambodia’s HEFs provide a robust architectural model for targeting subsidies and performance-based payments to the poorest quintiles, effectively shielding them from user fees. These existing mechanisms prove that targeted financial protection is feasible within the region and provide an empirical foundation for our policy recommendations.

Based on these findings, we propose four specific policy recommendations. First, countries should remove pre-diagnostic user fees by explicitly incorporating initial TB investigations into UHC benefit packages. Second, TB services should be designated as a public good within SHI systems, with exemptions from co-payments and reimbursement ceilings targeted particularly for poor, near-poor, and otherwise vulnerable populations. Third, purchasing reforms must align payment methods with patient-centred pathways and undergo continuous monitoring and revision based on provider responses to avoid perverse incentives and mitigate adverse outcomes. Finally, domestic financing transition plans should prioritise replacing donor-funded TB commodities with pooled public resources to ensure long-term sustainability.

This study has several strengths. To our knowledge, it is the first systematic comparative assessment in the Western Pacific Region examining how different national health financing architectures purchase and cover TB services across the full continuum of care. By combining structured survey data, qualitative interviews, and triangulation with policy and expenditure documents, the study provides an analytically grounded and regionally comparable typology of TB financing arrangements.

This study also has several limitations. First, data were self-reported by national focal points. While verified against literature, these data may reflect policy intent rather than de facto implementation at the facility level. Second, the cross-sectional nature of the survey captures a snapshot in time during active policy reforms, such as the payment reforms in China, which may evolve rapidly. Third, this study evaluates macro-level system architecture and policy design rather than observed patient-level expenditure. Our findings therefore identify structural risk factors for financial hardship; they do not quantify the actual magnitude of catastrophic costs at the household level. The approach is complementary to patient-level studies. Expenditure surveys quantify the magnitude of financial hardship; our assessment identifies its upstream structural origins. Fourth, this assessment focused strictly on health financing mechanisms for medical services. It deliberately excluded indirect economic burdens such as non-medical costs (e.g., transportation, nutrition) and income loss. Linking these design-level medical financing gaps with the broader social protection interventions required to address non-medical costs is a critical direction for future research. This dimension is comprehensively evaluated in our complementary regional analysis [18].

## Conclusion

This study shows that progress towards UHC in the Western Pacific Region has improved formal coverage of TB services but has not yet ensured comprehensive financial protection for TB medical care. Across diverse financing architectures, important gaps remain: pre-diagnostic services often generate out-of-pocket costs in budget-based systems, while co-payments and reimbursement limits persist in insurance-based systems. In several countries, services reported as fully covered are also heavily dependent on external donor financing, raising concerns about sustainability.

The findings suggest that financial protection for TB cannot be achieved by covering TB medicines alone. Financing systems need to purchase an integrated TB care pathway, including prevention, diagnostic workup, treatment, monitoring, and hospitalisation where clinically indicated. For national health service-type and government-subsidised systems, this requires closing the diagnostic gap and increasing flexibility in operational funding. For insurance-based systems, it requires adapting benefit design and provider payment mechanisms to the public health characteristics of TB, including exemptions from avoidable co-payments and careful monitoring of provider incentives.

Medical-service financing reforms are therefore a necessary foundation for improved TB financial protection under UHC. Future reforms should prioritise comprehensive coverage of essential TB medical services, sustainable domestic financing for donor-supported components, and purchasing arrangements that follow patients across the continuum of care.

## Supplementary Information

Supplementary material 1.

Supplementary material 2.

## Data Availability

De-identified, aggregated survey data supporting the findings of this study may be made available from the corresponding author upon reasonable request, subject to institutional approval. Individual interview transcripts are not publicly available due to confidentiality agreements with key informants and the sensitivity of country-level policy discussions.
