# Supplementary material for: Assessment of financial protection mechanisms for tuberculosis services in seven priority countries of the Western Pacific Region: a cross-sectional mixed-methods study
Source: Trop Med Health. 2026 Aug 6;54:137. doi: 10.1186/s41182-026-00979-7 (PMC13445896; doi:10.1186/s41182-026-00979-7)
Supplement: Supplementary file 1 [file 41182_2026_979_MOESM1_ESM.pdf]

Survey Questionnaire on Current TB Financial Protection Schemes in the Western Pacific Region

Thank you for participating in this survey on tuberculosis (TB) financial protection in Western Pacific Region.

- **Purpose**: We are aiming to assess the current financial protection schemes and policies available to TB prevention, diagnosis, and treatment in the Western Pacific region, specifically focusing on Cambodia, China, Lao PDR, Mongolia, Papua New Guinea, the Philippines, and Vietnam. Your insights are crucial for improving TB care and reducing the financial burden on patients and their families.
- **Instructions**: The survey questionnaire consists of six sections (Section A: General Question, Section B to G: Financial Schemes). Please complete all sections to the best of your knowledge. If a question does not apply to you, please leave it blank. For multiple-choice questions, select the most appropriate answer. Your honest and thorough responses are essential for a comprehensive evaluation of TB financial protection schemes in the Western Pacific Region.
- **Duration and Data Protection**: This survey will take approximately 25-30 minutes to complete. Your responses will be kept strictly confidential and used only for research purposes. No personally identifiable information will be shared.
- **Contact Information**: This survey is conducted by Korea University in collaboration with the World Health Organization Western Pacific Region Office. If you have any questions, please contact Dr. Hongjo Choi at hchoi.ku@gmail.com.

Thank you for your time and valuable input.

A. General question

Q1. Which of the following country are you from?

1. Cambodia
2. China
3. Lao People's Democratic Republic
4. Mongolia
5. Papua New Guinea
6. Philippines
7. Vietnam

Q2. Which of the following organization are you working for?

1. National TB Programme
2. Health financing or insurance agency or department
3. Other: _______________

In the following section, you will be asked about various scheme categories including:

Section	Categories	Description
B	National Health Service	A publicly funded healthcare system where the government owns and operates healthcare facilities and employs healthcare providers. financed through general taxation, it ensures healthcare is provided free or at minimal cost at the point at the point of service to all citizens.
C	National Health Insurance	A scheme where the state or society institutionally provides healthcare services to protect citizens' right to health. Primarily funded through premiums and partly through government subsidies, it aims to cover all citizens or a significant portion of the population.
D	Community Health Insurance	A scheme where community members pool funds to offset health costs, mainly targeted at informal sector workers, farmers, and/or low-income populations. Often voluntary, it may rely on shared responsibility within a community, with benefits tailored to local health needs and priorities.
E	Government Subsidies for Special Groups	A health financing mechanism that subsidizes healthcare costs for the poorest and most vulnerable populations. Funds are usually provided by governments, donors, or a combination, to ensure financial barriers do not prevent access to essential healthcare services.
F	NTP covered direct purchase	A scheme where the National TB Programme directly purchases, procures, and supplies TB-related commodities, including drugs and diagnostics, to health facilities and providers. Funding sources include the Global Fund to Fight AIDS, Tuberculosis and Malaria (Global Fund).
G	Other Scheme(s)	Any other healthcare financing or provision scheme not covered by the above categories

Your country's financial protection system may incorporate multiple schemes. Please review all categories and their definitions carefully, then select 'Yes' or 'No' for each scheme in the corresponding section. For instance, if your country has a mixed system including NHI, CHI, Health Equity Fund, and Global Fund, you should mark 'Yes' in sections C, D, E, and F, and proceed to answer the subsequent questions for each of these sections.

For each country, you will be asked to provide information on:

1. Applicability of each scheme to TB services
2. Primary funding sources
3. Eligibility criteria and entitlement
4. Claim mechanisms
5. Type of TB services covered
6. Extent of coverage for patients
7. Lowest level of facility providing each service

Please ensure you have access to detailed information about your country's TB-related financial protection schemes before proceeding.

Q3. Do you understand the content of the following section well enough to proceed?

1. Yes, I understand and am ready to proceed.
2. No, I need more information before continuing (If you select this option, please send an email to hchoi.ku@gmail.com with your detailed inquiries that need clarification.)

B. National Health Service

Q4. Is NHS (National Health Service) applied to TB services in your country?

1. No
2. Yes

Q4-1. What are the primary funding sources for NHS? (Select all that apply)

1. Compulsory contributions (e.g., payroll deductions)
2. Government revenues (e.g., taxes, social security funds)
3. External funding (e.g., donations, non-governmental organizations)
4. Employer and employee contributions
5. Other: _______________

Q4-2. What are the eligibility criteria for NHS? (Select all that apply)

1. Formal
2. Informal
3. Special group (vulnerable group); *e.g., people with disability, poor people, infants or children, adults aged 65 year or older, or pregnant women*
4. Other: _______________

Q4-2a. If 'Formal', what is the entitlement?

1. Mandatory
2. Voluntarily
3. Mixed

Q4-2b. If 'Informal', what is the entitlement?

1. Mandatory
2. Voluntarily
3. Mixed

Q4-2c. If 'Special group', what is the entitlement?

1. Mandatory
2. Voluntarily
3. Mixed

Q4-3. What is the claim mechanism for NHS? (Select all that apply)

1. Direct Payment (Out-Of-Pocket with reimbursement); Patients pay first, and claim reimbursement
2. Third-Party Billing (Provider to scheme); Providers bill the scheme, and patients pay little or nothing
3. No Claims Required (Fully subsidized services); Services are provided without payment or claims
4. Voucher or Prepaid Mechanism; Vouchers/cards are used, no claims needed
5. Integrated Public Systems; Services offered directly by public systems
6. Other: _______________

Q5. For each of the following services, please check 'Yes' if applicable, and provide information on the extent of coverage for patients and the lowest level of facility that provides these services:

Q5-1. Microscopy

1. No
2. Yes

Q5-1a. If 'Microscopy' is covered, what is the extent of coverage for patients?

1. No coverage
2. Less than 20% of total costs covered
3. 20~50% of total costs covered
4. More than 50% of total costs covered
5. Fully covered
6. Other: _______________

Q5-1b. What is the lowest level of facility that provides this service?

1. Lower-level facility than health center (e.g., health post)
2. Health center
3. District hospital
4. Regional (provincial) hospital
5. Tertiary hospital

Q5-2. Chest X-ray

1. No
2. Yes

Q5-2a. If 'Microscopy' is covered, what is the extent of coverage for patients?

1. No coverage
2. Less than 20% of total costs covered
3. 20~50% of total costs covered
4. More than 50% of total costs covered
5. Fully covered
6. Other: _______________

Q5-2b. What is the lowest level of facility that provides this service?

1. Lower-level facility than health center (e.g., health post)
2. Health center
3. District hospital
4. Regional (provincial) hospital
5. Tertiary hospital

Q5-3. Molecular TB diagnosis (NAAT: e.g., GeneXpert)

1. No
2. Yes

Q5-3a. If 'Molecular TB diagnosis (NAAT: e.g., GeneXpert)' is covered, what is the extent of coverage for patients?

1. No coverage
2. Less than 20% of total costs covered
3. 20~50% of total costs covered

4. More than 50% of total costs covered
5. Fully covered
6. Other: ______________

Q5-3b. What is the lowest level of facility that provides this service?

1. Lower-level facility than health center (e.g., health post)
2. Health center
3. District hospital
4. Regional (provincial) hospital
5. Tertiary hospital

Q5-4. LTBI diagnosis

1. No
2. Yes

Q5-4a. If 'LTBI diagnosis' is covered, what is the extent of coverage for patients?

1. No coverage
2. Less than 20% of total costs covered
3. 20~50% of total costs covered
4. More than 50% of total costs covered
5. Fully covered
6. Other: ______________

Q5-4b. What is the lowest level of facility that provides this service?

1. Lower-level facility than health center (e.g., health post)
2. Health center
3. District hospital
4. Regional (provincial) hospital
5. Tertiary hospital

Q5-5. Molecular DST (Drug-Susceptibility Test)

1. No
2. Yes

Q5-5a. If 'Molecular DST (Drug-Susceptibility Test)' is covered, what is the extent of coverage for patients?

1. No coverage
2. Less than 20% of total costs covered
3. 20~50% of total costs covered
4. More than 50% of total costs covered
5. Fully covered
6. Other: ______________

Q5-5b. What is the lowest level of facility that provides this service?

1. Lower-level facility than health center (e.g., health post)
2. Health center
3. District hospital
4. Regional (provincial) hospital
5. Tertiary hospital

Q5-6. Phenotypic DST (Drug-Susceptibility Test)

1. No
2. Yes

Q5-6a. If 'Phenotypic DST (Drug-Susceptibility Test)' is covered, what is the extent of coverage for patients?

1. No coverage
2. Less than 20% of total costs covered
3. 20~50% of total costs covered
4. More than 50% of total costs covered
5. Fully covered
6. Other: _______________

Q5-6b. What is the lowest level of facility that provides this service?

1. Lower-level facility than health center (e.g., health post)
2. Health center
3. District hospital
4. Regional (provincial) hospital
5. Tertiary hospital

Q5-7. First-line TB treatment

1. No
2. Yes

Q5-7a. If 'First-line TB treatment' is covered, what is the extent of coverage for patients?

1. No coverage
2. Less than 20% of total costs covered
3. 20~50% of total costs covered
4. More than 50% of total costs covered
5. Fully covered
6. Other: _______________

Q5-7b. What is the lowest level of facility that provides this service?

1. Lower-level facility than health center (e.g., health post)
2. Health center
3. District hospital
4. Regional (provincial) hospital
5. Tertiary hospital

Q5-8. First-line TB treatment

1. No
2. Yes

Q5-8a. If 'First-line TB treatment' is covered, what is the extent of coverage for patients?

1. No coverage
2. Less than 20% of total costs covered
3. 20~50% of total costs covered
4. More than 50% of total costs covered

5. Fully covered
6. Other: _______________

Q5-8b. What is the lowest level of facility that provides this service?

1. Lower-level facility than health center (e.g., health post)
2. Health center
3. District hospital
4. Regional (provincial) hospital
5. Tertiary hospital

Q5-9. Preventive treatment (LTBI)

1. No
2. Yes

Q5-9a. If 'Preventive treatment (LTBI)' is covered, what is the extent of coverage for patients?

1. No coverage
2. Less than 20% of total costs covered
3. 20~50% of total costs covered
4. More than 50% of total costs covered
5. Fully covered
6. Other: _______________

Q5-9b. What is the lowest level of facility that provides this service?

1. Lower-level facility than health center (e.g., health post)
2. Health center
3. District hospital
4. Regional (provincial) hospital
5. Tertiary hospital

Q5-10. Consultation (OPD)

1. No
2. Yes

Q5-10a. If 'Consultation (OPD)' is covered, what is the extent of coverage for patients?

1. No coverage
2. Less than 20% of total costs covered
3. 20~50% of total costs covered
4. More than 50% of total costs covered
5. Fully covered
6. Other: _______________

Q5-10b. What is the lowest level of facility that provides this service?

1. Lower-level facility than health center (e.g., health post)
2. Health center
3. District hospital
4. Regional (provincial) hospital
5. Tertiary hospital

Q5-11. Hospitalization (IPD)

1. No
2. Yes

Q5-11a. If 'Hospitalization (IPD)' is covered, what is the extent of coverage for patients?

1. No coverage
2. Less than 20% of total costs covered
3. 20~50% of total costs covered
4. More than 50% of total costs covered
5. Fully covered
6. Other: _______________

Q5-11b. What is the lowest level of facility that provides this service?

1. Lower-level facility than health center (e.g., health post)
2. Health center
3. District hospital
4. Regional (provincial) hospital
5. Tertiary hospital

Q5-12. DOT

1. No
2. Yes

Q5-12a. If 'DOT' is covered, what is the extent of coverage for patients?

1. No coverage
2. Less than 20% of total costs covered
3. 20~50% of total costs covered
4. More than 50% of total costs covered
5. Fully covered
6. Other: _______________

Q5-12b. What is the lowest level of facility that provides this service?

1. Lower-level facility than health center (e.g., health post)
2. Health center
3. District hospital
4. Regional (provincial) hospital
5. Tertiary hospital

Q5-13. If there are 'Others', please specify and provide details in a short answer. (If there is nothing, please leave it blank.): _______________

Q5-13a. If 'Others (the one specified above)' is covered, what is the extent of coverage for patients?

1. No coverage
2. Less than 20% of total costs covered
3. 20~50% of total costs covered
4. More than 50% of total costs covered
5. Fully covered
6. Other: _______________

Q5-13b. What is the lowest level of facility that provides this service?

1. Lower-level facility than health center (e.g., health post)
2. Health center
3. District hospital
4. Regional (provincial) hospital
5. Tertiary hospital

C. National Health Insurance

Q6. Is NHI (National Health Insurance) applied to TB services in your country?

1. No
2. Yes

Q6-1. What are the primary funding sources for NHI? (Select all that apply)

1. Compulsory contributions (e.g., payroll deductions)
2. Government revenues (e.g., taxes, social security funds)
3. External funding (e.g., donations, non-governmental organizations)
4. Employer and employee contributions
5. Other: _______________

Q6-2. What are the eligibility criteria for NHI? (Select all that apply)

1. Formal
2. Informal
3. Special group (vulnerable group); *e.g., people with disability, poor people, infants or children, adults aged 65 year or older, or pregnant women*
4. Other: _______________

Q6-2a. If 'Formal', what is the entitlement?

1. Mandatory
2. Voluntarily
3. Mixed

Q6-2b. If 'Informal', what is the entitlement?

1. Mandatory
2. Voluntarily
3. Mixed

Q6-2c. If 'Special group', what is the entitlement?

1. Mandatory
2. Voluntarily
3. Mixed

Q6-3. What is the claim mechanism for NHI? (Select all that apply)

1. Direct Payment (Out-Of-Pocket with reimbursement); Patients pay first, and claim reimbursement
2. Third-Party Billing (Provider to scheme); Providers bill the scheme, and patients pay little or nothing
3. No Claims Required (Fully subsidized services); Services are provided without payment or claims
4. Voucher or Prepaid Mechanism; Vouchers/cards are used, no claims needed
5. Integrated Public Systems; Services offered directly by public systems
6. Other: _______________

Q7. For each of the following services, please check 'Yes' if applicable, and provide information on the extent of coverage for patients and the lowest level of facility that provides these services:

Q7-1. Microscopy

1. No
2. Yes

Q7-1a. If 'Microscopy' is covered, what is the extent of coverage for patients?

1. No coverage
2. Less than 20% of total costs covered
3. 20~50% of total costs covered
4. More than 50% of total costs covered
5. Fully covered
6. Other: _______________

Q7-1b. What is the lowest level of facility that provides this service?

1. Lower-level facility than health center (e.g., health post)
2. Health center
3. District hospital
4. Regional (provincial) hospital
5. Tertiary hospital

Q7-2. Chest X-ray

1. No
2. Yes

Q7-2a. If 'Microscopy' is covered, what is the extent of coverage for patients?

1. No coverage
2. Less than 20% of total costs covered
3. 20~50% of total costs covered
4. More than 50% of total costs covered
5. Fully covered
6. Other: _______________

Q7-2b. What is the lowest level of facility that provides this service?

1. Lower-level facility than health center (e.g., health post)
2. Health center
3. District hospital
4. Regional (provincial) hospital
5. Tertiary hospital

Q7-3. Molecular TB diagnosis (NAAT: e.g., GeneXpert)

1. No
2. Yes

Q7-3a. If 'Molecular TB diagnosis (NAAT: e.g., GeneXpert)' is covered, what is the extent of coverage for patients?

1. No coverage
2. Less than 20% of total costs covered
3. 20~50% of total costs covered

4. More than 50% of total costs covered
5. Fully covered
6. Other: _______________

Q7-3b. What is the lowest level of facility that provides this service?

1. Lower-level facility than health center (e.g., health post)
2. Health center
3. District hospital
4. Regional (provincial) hospital
5. Tertiary hospital

Q7-4. LTBI diagnosis

1. No
2. Yes

Q7-4a. If 'LTBI diagnosis' is covered, what is the extent of coverage for patients?

1. No coverage
2. Less than 20% of total costs covered
3. 20~50% of total costs covered
4. More than 50% of total costs covered
5. Fully covered
6. Other: _______________

Q7-4b. What is the lowest level of facility that provides this service?

1. Lower-level facility than health center (e.g., health post)
2. Health center
3. District hospital
4. Regional (provincial) hospital
5. Tertiary hospital

Q7-5. Molecular DST (Drug-Susceptibility Test)

1. No
2. Yes

Q7-5a. If 'Molecular DST (Drug-Susceptibility Test)' is covered, what is the extent of coverage for patients?

1. No coverage
2. Less than 20% of total costs covered
3. 20~50% of total costs covered
4. More than 50% of total costs covered
5. Fully covered
6. Other: _______________

Q7-5b. What is the lowest level of facility that provides this service?

1. Lower-level facility than health center (e.g., health post)
2. Health center
3. District hospital
4. Regional (provincial) hospital
5. Tertiary hospital

Q7-6. Phenotypic DST (Drug-Susceptibility Test)

1. No
2. Yes

Q7-6a. If 'Phenotypic DST (Drug-Susceptibility Test)' is covered, what is the extent of coverage for patients?

1. No coverage
2. Less than 20% of total costs covered
3. 20~50% of total costs covered
4. More than 50% of total costs covered
5. Fully covered
6. Other: _______________

Q7-6b. What is the lowest level of facility that provides this service?

1. Lower-level facility than health center (e.g., health post)
2. Health center
3. District hospital
4. Regional (provincial) hospital
5. Tertiary hospital

Q7-7. First-line TB treatment

1. No
2. Yes

Q7-7a. If 'First-line TB treatment' is covered, what is the extent of coverage for patients?

1. No coverage
2. Less than 20% of total costs covered
3. 20~50% of total costs covered
4. More than 50% of total costs covered
5. Fully covered
6. Other: _______________

Q7-7b. What is the lowest level of facility that provides this service?

1. Lower-level facility than health center (e.g., health post)
2. Health center
3. District hospital
4. Regional (provincial) hospital
5. Tertiary hospital

Q7-8. First-line TB treatment

1. No
2. Yes

Q7-8a. If 'First-line TB treatment' is covered, what is the extent of coverage for patients?

1. No coverage
2. Less than 20% of total costs covered
3. 20~50% of total costs covered
4. More than 50% of total costs covered

5. Fully covered
6. Other: _______________

Q7-8b. What is the lowest level of facility that provides this service?

1. Lower-level facility than health center (e.g., health post)
2. Health center
3. District hospital
4. Regional (provincial) hospital
5. Tertiary hospital

Q7-9. Preventive treatment (LTBI)

1. No
2. Yes

Q7-9a. If 'Preventive treatment (LTBI)' is covered, what is the extent of coverage for patients?

1. No coverage
2. Less than 20% of total costs covered
3. 20~50% of total costs covered
4. More than 50% of total costs covered
5. Fully covered
6. Other: _______________

Q7-9b. What is the lowest level of facility that provides this service?

1. Lower-level facility than health center (e.g., health post)
2. Health center
3. District hospital
4. Regional (provincial) hospital
5. Tertiary hospital

Q7-10. Consultation (OPD)

1. No
2. Yes

Q7-10a. If 'Consultation (OPD)' is covered, what is the extent of coverage for patients?

1. No coverage
2. Less than 20% of total costs covered
3. 20~50% of total costs covered
4. More than 50% of total costs covered
5. Fully covered
6. Other: _______________

Q7-10b. What is the lowest level of facility that provides this service?

1. Lower-level facility than health center (e.g., health post)
2. Health center
3. District hospital
4. Regional (provincial) hospital
5. Tertiary hospital

Q7-11. Hospitalization (IPD)

1. No
2. Yes

Q7-11a. If 'Hospitalization (IPD)' is covered, what is the extent of coverage for patients?

1. No coverage
2. Less than 20% of total costs covered
3. 20~50% of total costs covered
4. More than 50% of total costs covered
5. Fully covered
6. Other: _______________

Q7-11b. What is the lowest level of facility that provides this service?

1. Lower-level facility than health center (e.g., health post)
2. Health center
3. District hospital
4. Regional (provincial) hospital
5. Tertiary hospital

Q7-12. DOT

1. No
2. Yes

Q7-12a. If 'DOT' is covered, what is the extent of coverage for patients?

1. No coverage
2. Less than 20% of total costs covered
3. 20~50% of total costs covered
4. More than 50% of total costs covered
5. Fully covered
6. Other: _______________

Q7-12b. What is the lowest level of facility that provides this service?

1. Lower-level facility than health center (e.g., health post)
2. Health center
3. District hospital
4. Regional (provincial) hospital
5. Tertiary hospital

Q7-13. If there are 'Others', please specify and provide details in a short answer. (If there is nothing, please leave it blank.): _______________

Q7-13a. If 'Others (the one specified above)' is covered, what is the extent of coverage for patients?

1. No coverage
2. Less than 20% of total costs covered
3. 20~50% of total costs covered
4. More than 50% of total costs covered
5. Fully covered
6. Other: _______________

Q7-13b. What is the lowest level of facility that provides this service?

1. Lower-level facility than health center (e.g., health post)
2. Health center
3. District hospital
4. Regional (provincial) hospital
5. Tertiary hospital

D. Community Health Insurance

Q8. Is CHI (Community Health Insurance) applied to TB services in your country?

1. No
2. Yes

Q8-1. What are the primary funding sources for CHI? (Select all that apply)

1. Compulsory contributions (e.g., payroll deductions)
2. Government revenues (e.g., taxes, social security funds)
3. External funding (e.g., donations, non-governmental organizations)
4. Employer and employee contributions
5. Other: _______________

Q8-2. What are the eligibility criteria for CHI? (Select all that apply)

1. Formal
2. Informal
3. Special group (vulnerable group); *e.g., people with disability, poor people, infants or children, adults aged 65 year or older, or pregnant women*
4. Other: _______________

Q8-2a. If 'Formal', what is the entitlement?

1. Mandatory
2. Voluntarily
3. Mixed

Q8-2b. If 'Informal', what is the entitlement?

1. Mandatory
2. Voluntarily
3. Mixed

Q8-2c. If 'Special group', what is the entitlement?

1. Mandatory
2. Voluntarily
3. Mixed

Q8-3. What is the claim mechanism for CHI? (Select all that apply)

1. Direct Payment (Out-Of-Pocket with reimbursement); Patients pay first, and claim reimbursement
2. Third-Party Billing (Provider to scheme); Providers bill the scheme, and patients pay little or nothing
3. No Claims Required (Fully subsidized services); Services are provided without payment or claims
4. Voucher or Prepaid Mechanism; Vouchers/cards are used, no claims needed
5. Integrated Public Systems; Services offered directly by public systems
6. Other: _______________

Q9. For each of the following services, please check 'Yes' if applicable, and provide information on the extent of coverage for patients and the lowest level of facility that provides these services:

Q9-1. Microscopy

1. No
2. Yes

Q9-1a. If 'Microscopy' is covered, what is the extent of coverage for patients?

1. No coverage
2. Less than 20% of total costs covered
3. 20~50% of total costs covered
4. More than 50% of total costs covered
5. Fully covered
6. Other: ______________

Q9-1b. What is the lowest level of facility that provides this service?

1. Lower-level facility than health center (e.g., health post)
2. Health center
3. District hospital
4. Regional (provincial) hospital
5. Tertiary hospital

Q9-2. Chest X-ray

1. No
2. Yes

Q9-2a. If 'Microscopy' is covered, what is the extent of coverage for patients?

1. No coverage
2. Less than 20% of total costs covered
3. 20~50% of total costs covered
4. More than 50% of total costs covered
5. Fully covered
6. Other: ______________

Q9-2b. What is the lowest level of facility that provides this service?

1. Lower-level facility than health center (e.g., health post)
2. Health center
3. District hospital
4. Regional (provincial) hospital
5. Tertiary hospital

Q9-3. Molecular TB diagnosis (NAAT: e.g., GeneXpert)

1. No
2. Yes

Q9-3a. If 'Molecular TB diagnosis (NAAT: e.g., GeneXpert)' is covered, what is the extent of coverage for patients?

1. No coverage
2. Less than 20% of total costs covered
3. 20~50% of total costs covered

4. More than 50% of total costs covered
5. Fully covered
6. Other: _______________

Q9-3b. What is the lowest level of facility that provides this service?

1. Lower-level facility than health center (e.g., health post)
2. Health center
3. District hospital
4. Regional (provincial) hospital
5. Tertiary hospital

Q9-4. LTBI diagnosis

1. No
2. Yes

Q9-4a. If 'LTBI diagnosis' is covered, what is the extent of coverage for patients?

1. No coverage
2. Less than 20% of total costs covered
3. 20~50% of total costs covered
4. More than 50% of total costs covered
5. Fully covered
6. Other: _______________

Q9-4b. What is the lowest level of facility that provides this service?

1. Lower-level facility than health center (e.g., health post)
2. Health center
3. District hospital
4. Regional (provincial) hospital
5. Tertiary hospital

Q9-5. Molecular DST (Drug-Susceptibility Test)

1. No
2. Yes

Q9-5a. If 'Molecular DST (Drug-Susceptibility Test)' is covered, what is the extent of coverage for patients?

1. No coverage
2. Less than 20% of total costs covered
3. 20~50% of total costs covered
4. More than 50% of total costs covered
5. Fully covered
6. Other: _______________

Q9-5b. What is the lowest level of facility that provides this service?

1. Lower-level facility than health center (e.g., health post)
2. Health center
3. District hospital
4. Regional (provincial) hospital
5. Tertiary hospital

Q9-6. Phenotypic DST (Drug-Susceptibility Test)

1. No
2. Yes

Q9-6a. If 'Phenotypic DST (Drug-Susceptibility Test)' is covered, what is the extent of coverage for patients?

1. No coverage
2. Less than 20% of total costs covered
3. 20~50% of total costs covered
4. More than 50% of total costs covered
5. Fully covered
6. Other: _______________

Q9-6b. What is the lowest level of facility that provides this service?

1. Lower-level facility than health center (e.g., health post)
2. Health center
3. District hospital
4. Regional (provincial) hospital
5. Tertiary hospital

Q9-7. First-line TB treatment

1. No
2. Yes

Q9-7a. If 'First-line TB treatment' is covered, what is the extent of coverage for patients?

1. No coverage
2. Less than 20% of total costs covered
3. 20~50% of total costs covered
4. More than 50% of total costs covered
5. Fully covered
6. Other: _______________

Q9-7b. What is the lowest level of facility that provides this service?

1. Lower-level facility than health center (e.g., health post)
2. Health center
3. District hospital
4. Regional (provincial) hospital
5. Tertiary hospital

Q9-8. First-line TB treatment

1. No
2. Yes

Q9-8a. If 'First-line TB treatment' is covered, what is the extent of coverage for patients?

1. No coverage
2. Less than 20% of total costs covered
3. 20~50% of total costs covered
4. More than 50% of total costs covered

5. Fully covered
6. Other: _______________

Q9-8b. What is the lowest level of facility that provides this service?

1. Lower-level facility than health center (e.g., health post)
2. Health center
3. District hospital
4. Regional (provincial) hospital
5. Tertiary hospital

Q9-9. Preventive treatment (LTBI)

1. No
2. Yes

Q9-9a. If 'Preventive treatment (LTBI)' is covered, what is the extent of coverage for patients?

1. No coverage
2. Less than 20% of total costs covered
3. 20~50% of total costs covered
4. More than 50% of total costs covered
5. Fully covered
6. Other: _______________

Q9-9b. What is the lowest level of facility that provides this service?

1. Lower-level facility than health center (e.g., health post)
2. Health center
3. District hospital
4. Regional (provincial) hospital
5. Tertiary hospital

Q9-10. Consultation (OPD)

1. No
2. Yes

Q9-10a. If 'Consultation (OPD)' is covered, what is the extent of coverage for patients?

1. No coverage
2. Less than 20% of total costs covered
3. 20~50% of total costs covered
4. More than 50% of total costs covered
5. Fully covered
6. Other: _______________

Q9-10b. What is the lowest level of facility that provides this service?

1. Lower-level facility than health center (e.g., health post)
2. Health center
3. District hospital
4. Regional (provincial) hospital
5. Tertiary hospital

Q9-11. Hospitalization (IPD)

1. No
2. Yes

Q9-11a. If 'Hospitalization (IPD)' is covered, what is the extent of coverage for patients?

1. No coverage
2. Less than 20% of total costs covered
3. 20~50% of total costs covered
4. More than 50% of total costs covered
5. Fully covered
6. Other: _______________

Q9-11b. What is the lowest level of facility that provides this service?

1. Lower-level facility than health center (e.g., health post)
2. Health center
3. District hospital
4. Regional (provincial) hospital
5. Tertiary hospital

Q9-12. DOT

1. No
2. Yes

Q9-12a. If 'DOT' is covered, what is the extent of coverage for patients?

1. No coverage
2. Less than 20% of total costs covered
3. 20~50% of total costs covered
4. More than 50% of total costs covered
5. Fully covered
6. Other: _______________

Q9-12b. What is the lowest level of facility that provides this service?

1. Lower-level facility than health center (e.g., health post)
2. Health center
3. District hospital
4. Regional (provincial) hospital
5. Tertiary hospital

Q9-13. If there are 'Others', please specify and provide details in a short answer. (If there is nothing, please leave it blank.): _______________

Q9-13a. If 'Others (the one specified above)' is covered, what is the extent of coverage for patients?

1. No coverage
2. Less than 20% of total costs covered
3. 20~50% of total costs covered
4. More than 50% of total costs covered
5. Fully covered
6. Other: _______________

Q9-13b. What is the lowest level of facility that provides this service?

1. Lower-level facility than health center (e.g., health post)
2. Health center
3. District hospital
4. Regional (provincial) hospital
5. Tertiary hospital

E. Government Subsidies for Special Group (e.g., Health Equity Fund)

Q10. Is Government Subsidies for Special Groups applied to TB services in your country?

1. No
2. Yes

Q10-1. What are the primary funding sources for the Government Subsidies for Special Groups? (Select all that apply)

1. Compulsory contributions (e.g., payroll deductions)
2. Government revenues (e.g., taxes, social security funds)
3. External funding (e.g., donations, non-governmental organizations)
4. Employer and employee contributions
5. Other: _______________

Q10-2. What are the eligibility criteria for the Government Subsidies for Special Groups? (Select all that apply)

1. Formal
2. Informal
3. Special group (vulnerable group); *e.g., people with disability, poor people, infants or children, adults aged 65 year or older, or pregnant women*
4. Other: _______________

Q10-2a. If 'Formal', what is the entitlement?

1. Mandatory
2. Voluntarily
3. Mixed

Q10-2b. If 'Informal', what is the entitlement?

1. Mandatory
2. Voluntarily
3. Mixed

Q10-2c. If 'Special group', what is the entitlement?

1. Mandatory
2. Voluntarily
3. Mixed

Q10-3. What is the claim mechanism for the Government Subsidies for Special Groups? (Select all that apply)

1. Direct Payment (Out-Of-Pocket with reimbursement); Patients pay first, and claim reimbursement
2. Third-Party Billing (Provider to scheme); Providers bill the scheme, and patients pay little or nothing
3. No Claims Required (Fully subsidized services); Services are provided without payment or claims
4. Voucher or Prepaid Mechanism; Vouchers/cards are used, no claims needed
5. Integrated Public Systems; Services offered directly by public systems

6. Other: _______________

Q11. For each of the following services, please check 'Yes' if applicable, and provide information on the extent of coverage for patients and the lowest level of facility that provides these services:

Q11-1. Microscopy

1. No
2. Yes

Q11-1a. If 'Microscopy' is covered, what is the extent of coverage for patients?

1. No coverage
2. Less than 20% of total costs covered
3. 20~50% of total costs covered
4. More than 50% of total costs covered
5. Fully covered
6. Other: _______________

Q11-1b. What is the lowest level of facility that provides this service?

1. Lower-level facility than health center (e.g., health post)
2. Health center
3. District hospital
4. Regional (provincial) hospital
5. Tertiary hospital

Q11-2. Chest X-ray

1. No
2. Yes

Q11-2a. If 'Microscopy' is covered, what is the extent of coverage for patients?

1. No coverage
2. Less than 20% of total costs covered
3. 20~50% of total costs covered
4. More than 50% of total costs covered
5. Fully covered
6. Other: _______________

Q11-2b. What is the lowest level of facility that provides this service?

1. Lower-level facility than health center (e.g., health post)
2. Health center
3. District hospital
4. Regional (provincial) hospital
5. Tertiary hospital

Q11-3. Molecular TB diagnosis (NAAT: e.g., GeneXpert)

1. No
2. Yes

Q11-3a. If 'Molecular TB diagnosis (NAAT: e.g., GeneXpert)' is covered, what is the extent of coverage for patients?

1. No coverage
2. Less than 20% of total costs covered
3. 20~50% of total costs covered
4. More than 50% of total costs covered
5. Fully covered
6. Other: _______________

Q11-3b. What is the lowest level of facility that provides this service?

1. Lower-level facility than health center (e.g., health post)
2. Health center
3. District hospital
4. Regional (provincial) hospital
5. Tertiary hospital

Q11-4. LTBI diagnosis

1. No
2. Yes

Q11-4a. If 'LTBI diagnosis' is covered, what is the extent of coverage for patients?

1. No coverage
2. Less than 20% of total costs covered
3. 20~50% of total costs covered
4. More than 50% of total costs covered
5. Fully covered
6. Other: _______________

Q11-4b. What is the lowest level of facility that provides this service?

1. Lower-level facility than health center (e.g., health post)
2. Health center
3. District hospital
4. Regional (provincial) hospital
5. Tertiary hospital

Q11-5. Molecular DST (Drug-Susceptibility Test)

1. No
2. Yes

Q11-5a. If 'Molecular DST (Drug-Susceptibility Test)' is covered, what is the extent of coverage for patients?

1. No coverage
2. Less than 20% of total costs covered
3. 20~50% of total costs covered
4. More than 50% of total costs covered
5. Fully covered
6. Other: _______________

Q11-5b. What is the lowest level of facility that provides this service?

6. Lower-level facility than health center (e.g., health post)
7. Health center
8. District hospital

9. Regional (provincial) hospital
10. Tertiary hospital

Q11-6. Phenotypic DST (Drug-Susceptibility Test)

1. No
2. Yes

Q11-6a. If 'Phenotypic DST (Drug-Susceptibility Test)' is covered, what is the extent of coverage for patients?

1. No coverage
2. Less than 20% of total costs covered
3. 20~50% of total costs covered
4. More than 50% of total costs covered
5. Fully covered
6. Other: _______________

Q11-6b. What is the lowest level of facility that provides this service?

1. Lower-level facility than health center (e.g., health post)
2. Health center
3. District hospital
4. Regional (provincial) hospital
5. Tertiary hospital

Q11-7. First-line TB treatment

1. No
2. Yes

Q11-7a. If 'First-line TB treatment' is covered, what is the extent of coverage for patients?

1. No coverage
2. Less than 20% of total costs covered
3. 20~50% of total costs covered
4. More than 50% of total costs covered
5. Fully covered
6. Other: _______________

Q11-7b. What is the lowest level of facility that provides this service?

1. Lower-level facility than health center (e.g., health post)
2. Health center
3. District hospital
4. Regional (provincial) hospital
5. Tertiary hospital

Q11-8. First-line TB treatment

1. No
2. Yes

Q11-8a. If 'First-line TB treatment' is covered, what is the extent of coverage for patients?

1. No coverage

2. Less than 20% of total costs covered
3. 20~50% of total costs covered
4. More than 50% of total costs covered
5. Fully covered
6. Other: _______________

Q11-8b. What is the lowest level of facility that provides this service?

1. Lower-level facility than health center (e.g., health post)
2. Health center
3. District hospital
4. Regional (provincial) hospital
5. Tertiary hospital

Q11-9. Preventive treatment (LTBI)

1. No
2. Yes

Q11-9a. If 'Preventive treatment (LTBI)' is covered, what is the extent of coverage for patients?

1. No coverage
2. Less than 20% of total costs covered
3. 20~50% of total costs covered
4. More than 50% of total costs covered
5. Fully covered
6. Other: _______________

Q11-9b. What is the lowest level of facility that provides this service?

1. Lower-level facility than health center (e.g., health post)
2. Health center
3. District hospital
4. Regional (provincial) hospital
5. Tertiary hospital

Q11-10. Consultation (OPD)

1. No
2. Yes

Q11-10a. If 'Consultation (OPD)' is covered, what is the extent of coverage for patients?

1. No coverage
2. Less than 20% of total costs covered
3. 20~50% of total costs covered
4. More than 50% of total costs covered
5. Fully covered
6. Other: _______________

Q11-10b. What is the lowest level of facility that provides this service?

1. Lower-level facility than health center (e.g., health post)
2. Health center
3. District hospital
4. Regional (provincial) hospital

5. Tertiary hospital

Q11-11. Hospitalization (IPD)

1. No
2. Yes

Q11-11a. If 'Hospitalization (IPD)' is covered, what is the extent of coverage for patients?

1. No coverage
2. Less than 20% of total costs covered
3. 20~50% of total costs covered
4. More than 50% of total costs covered
5. Fully covered
6. Other: _______________

Q11-11b. What is the lowest level of facility that provides this service?

1. Lower-level facility than health center (e.g., health post)
2. Health center
3. District hospital
4. Regional (provincial) hospital
5. Tertiary hospital

Q11-12. DOT

1. No
2. Yes

Q11-12a. If 'DOT' is covered, what is the extent of coverage for patients?

1. No coverage
2. Less than 20% of total costs covered
3. 20~50% of total costs covered
4. More than 50% of total costs covered
5. Fully covered
6. Other: _______________

Q11-12b. What is the lowest level of facility that provides this service?

1. Lower-level facility than health center (e.g., health post)
2. Health center
3. District hospital
4. Regional (provincial) hospital
5. Tertiary hospital

Q11-13. If there are 'Others', please specify and provide details in a short answer. (If there is nothing, please leave it blank.): _______________

Q11-13a. If 'Others (the one specified above)' is covered, what is the extent of coverage for patients?

1. No coverage
2. Less than 20% of total costs covered
3. 20~50% of total costs covered
4. More than 50% of total costs covered
5. Fully covered

6. Other: _______________

Q11-13b. What is the lowest level of facility that provides this service?

1. Lower-level facility than health center (e.g., health post)
2. Health center
3. District hospital
4. Regional (provincial) hospital
5. Tertiary hospital

F. NTP covered direct purchase (e.g., Global Fund)

Q12. Is NTP covered direct purchase (such as Global Fund) applied to TB services in your country?

1. No
2. Yes

Q12-1. What are the primary funding sources for the NTP covered direct purchase? (Select all that apply)

1. Compulsory contributions (e.g., payroll deductions)
2. Government revenues (e.g., taxes, social security funds)
3. External funding (e.g., donations, non-governmental organizations)
4. Employer and employee contributions
5. Other: _______________

Q12-2. What are the eligibility criteria for the NTP covered direct purchase? (Select all that apply)

1. Formal
2. Informal
3. Special group (vulnerable group); *e.g., people with disability, poor people, infants or children, adults aged 65 year or older, or pregnant women*
4. Other: _______________

Q12-2a. If 'Formal', what is the entitlement?

1. Mandatory
2. Voluntarily
3. Mixed

Q12-2b. If 'Informal', what is the entitlement?

1. Mandatory
2. Voluntarily
3. Mixed

Q12-2c. If 'Special group', what is the entitlement?

1. Mandatory
2. Voluntarily
3. Mixed

Q12-3. What is the claim mechanism for the NTP covered direct purchase? (Select all that apply)

1. Direct Payment (Out-Of-Pocket with reimbursement); Patients pay first, and claim reimbursement
2. Third-Party Billing (Provider to scheme); Providers bill the scheme, and patients pay little or nothing
3. No Claims Required (Fully subsidized services); Services are provided without payment or claims
4. Voucher or Prepaid Mechanism; Vouchers/cards are used, no claims needed
5. Integrated Public Systems; Services offered directly by public systems
6. Other: _______________

Q13. For each of the following services, please check 'Yes' if applicable, and provide information on the extent of coverage for patients and the lowest level of facility that provides these services:

Q13-1. Microscopy

1. No
2. Yes

Q13-1a. If 'Microscopy' is covered, what is the extent of coverage for patients?

1. No coverage
2. Less than 20% of total costs covered
3. 20~50% of total costs covered
4. More than 50% of total costs covered
5. Fully covered
6. Other: _______________

Q13-1b. What is the lowest level of facility that provides this service?

1. Lower-level facility than health center (e.g., health post)
2. Health center
3. District hospital
4. Regional (provincial) hospital
5. Tertiary hospital

Q13-2. Chest X-ray

1. No
2. Yes

Q13-2a. If 'Microscopy' is covered, what is the extent of coverage for patients?

1. No coverage
2. Less than 20% of total costs covered
3. 20~50% of total costs covered
4. More than 50% of total costs covered
5. Fully covered
6. Other: _______________

Q13-2b. What is the lowest level of facility that provides this service?

1. Lower-level facility than health center (e.g., health post)
2. Health center
3. District hospital
4. Regional (provincial) hospital
5. Tertiary hospital

Q13-3. Molecular TB diagnosis (NAAT: e.g., GeneXpert)

1. No
2. Yes

Q13-3a. If 'Molecular TB diagnosis (NAAT: e.g., GeneXpert)' is covered, what is the extent of coverage for patients?

1. No coverage

2. Less than 20% of total costs covered
3. 20~50% of total costs covered
4. More than 50% of total costs covered
5. Fully covered
6. Other: _______________

Q13-3b. What is the lowest level of facility that provides this service?

1. Lower-level facility than health center (e.g., health post)
2. Health center
3. District hospital
4. Regional (provincial) hospital
5. Tertiary hospital

Q13-4. LTBI diagnosis

1. No
2. Yes

Q13-4a. If 'LTBI diagnosis' is covered, what is the extent of coverage for patients?

1. No coverage
2. Less than 20% of total costs covered
3. 20~50% of total costs covered
4. More than 50% of total costs covered
5. Fully covered
6. Other: _______________

Q13-4b. What is the lowest level of facility that provides this service?

1. Lower-level facility than health center (e.g., health post)
2. Health center
3. District hospital
4. Regional (provincial) hospital
5. Tertiary hospital

Q13-5. Molecular DST (Drug-Susceptibility Test)

1. No
2. Yes

Q13-5a. If 'Molecular DST (Drug-Susceptibility Test)' is covered, what is the extent of coverage for patients?

1. No coverage
2. Less than 20% of total costs covered
3. 20~50% of total costs covered
4. More than 50% of total costs covered
5. Fully covered
6. Other: _______________

Q13-5b. What is the lowest level of facility that provides this service?

1. Lower-level facility than health center (e.g., health post)
2. Health center
3. District hospital
4. Regional (provincial) hospital

5. Tertiary hospital

Q13-6. Phenotypic DST (Drug-Susceptibility Test)

1. No
2. Yes

Q13-6a. If 'Phenotypic DST (Drug-Susceptibility Test)' is covered, what is the extent of coverage for patients?

1. No coverage
2. Less than 20% of total costs covered
3. 20~50% of total costs covered
4. More than 50% of total costs covered
5. Fully covered
6. Other: _______________

Q13-6b. What is the lowest level of facility that provides this service?

1. Lower-level facility than health center (e.g., health post)
2. Health center
3. District hospital
4. Regional (provincial) hospital
5. Tertiary hospital

Q13-7. First-line TB treatment

1. No
2. Yes

Q13-7a. If 'First-line TB treatment' is covered, what is the extent of coverage for patients?

1. No coverage
2. Less than 20% of total costs covered
3. 20~50% of total costs covered
4. More than 50% of total costs covered
5. Fully covered
6. Other: _______________

Q13-7b. What is the lowest level of facility that provides this service?

1. Lower-level facility than health center (e.g., health post)
2. Health center
3. District hospital
4. Regional (provincial) hospital
5. Tertiary hospital

Q13-8. First-line TB treatment

1. No
2. Yes

Q13-8a. If 'First-line TB treatment' is covered, what is the extent of coverage for patients?

1. No coverage
2. Less than 20% of total costs covered

3. 20~50% of total costs covered
4. More than 50% of total costs covered
5. Fully covered
6. Other: ______________

Q13-8b. What is the lowest level of facility that provides this service?

1. Lower-level facility than health center (e.g., health post)
2. Health center
3. District hospital
4. Regional (provincial) hospital
5. Tertiary hospital

Q13-9. Preventive treatment (LTBI)

1. No
2. Yes

Q13-9a. If 'Preventive treatment (LTBI)' is covered, what is the extent of coverage for patients?

1. No coverage
2. Less than 20% of total costs covered
3. 20~50% of total costs covered
4. More than 50% of total costs covered
5. Fully covered
6. Other: ______________

Q13-9b. What is the lowest level of facility that provides this service?

1. Lower-level facility than health center (e.g., health post)
2. Health center
3. District hospital
4. Regional (provincial) hospital
5. Tertiary hospital

Q13-10. Consultation (OPD)

1. No
2. Yes

Q13-10a. If 'Consultation (OPD)' is covered, what is the extent of coverage for patients?

1. No coverage
2. Less than 20% of total costs covered
3. 20~50% of total costs covered
4. More than 50% of total costs covered
5. Fully covered
6. Other: ______________

Q13-10b. What is the lowest level of facility that provides this service?

1. Lower-level facility than health center (e.g., health post)
2. Health center
3. District hospital
4. Regional (provincial) hospital
5. Tertiary hospital

Q13-11. Hospitalization (IPD)

1. No
2. Yes

Q13-11a. If 'Hospitalization (IPD)' is covered, what is the extent of coverage for patients?

1. No coverage
2. Less than 20% of total costs covered
3. 20~50% of total costs covered
4. More than 50% of total costs covered
5. Fully covered
6. Other: _______________

Q13-11b. What is the lowest level of facility that provides this service?

1. Lower-level facility than health center (e.g., health post)
2. Health center
3. District hospital
4. Regional (provincial) hospital
5. Tertiary hospital

Q13-12. DOT

1. No
2. Yes

Q13-12a. If 'DOT' is covered, what is the extent of coverage for patients?

1. No coverage
2. Less than 20% of total costs covered
3. 20~50% of total costs covered
4. More than 50% of total costs covered
5. Fully covered
6. Other: _______________

Q13-12b. What is the lowest level of facility that provides this service?

1. Lower-level facility than health center (e.g., health post)
2. Health center
3. District hospital
4. Regional (provincial) hospital
5. Tertiary hospital

Q13-13. If there are 'Others', please specify and provide details in a short answer. (If there is nothing, please leave it blank.): _______________

Q13-13a. If 'Others (the one specified above)' is covered, what is the extent of coverage for patients?

1. No coverage
2. Less than 20% of total costs covered
3. 20~50% of total costs covered
4. More than 50% of total costs covered
5. Fully covered
6. Other: _______________

Q13-13b. What is the lowest level of facility that provides this service?

1. Lower-level facility than health center (e.g., health post)
2. Health center
3. District hospital
4. Regional (provincial) hospital
5. Tertiary hospital

G. Other Scheme(s)

Q14. Are there any other financial scheme(s) applied to TB services in your country?

1. No
2. Yes

Q14-1. If 'Yes', please specify the name(s) of the scheme(s). ________________

Q14-2. What are the primary funding sources for the scheme that you specified? (Select all that apply)

1. Compulsory contributions (e.g., payroll deductions)
2. Government revenues (e.g., taxes, social security funds)
3. External funding (e.g., donations, non-governmental organizations)
4. Employer and employee contributions
5. Other: _______________

Q14-3. What are the eligibility criteria for the scheme that you specified? (Select all that apply)

1. Formal
2. Informal
3. Special group (vulnerable group); *e.g., people with disability, poor people, infants or children, adults aged 65 year or older, or pregnant women*
4. Other: _______________

Q14-3a. If 'Formal', what is the entitlement?

1. Mandatory
2. Voluntarily
3. Mixed

Q14-3b. If 'Informal', what is the entitlement?

1. Mandatory
2. Voluntarily
3. Mixed

Q14-3c. If 'Special group', what is the entitlement?

1. Mandatory
2. Voluntarily
3. Mixed

Q14-4. What is the claim mechanism for the scheme that you specified? (Select all that apply)

1. Direct Payment (Out-Of-Pocket with reimbursement); Patients pay first, and claim reimbursement
2. Third-Party Billing (Provider to scheme); Providers bill the scheme, and patients pay little or nothing
3. No Claims Required (Fully subsidized services); Services are provided without payment or claims
4. Voucher or Prepaid Mechanism; Vouchers/cards are used, no claims needed
5. Integrated Public Systems; Services offered directly by public systems
6. Other: _______________

Q15. For each of the following services, please check 'Yes' if applicable, and provide information on the extent of coverage for patients and the lowest level of facility that provides these services:

Q15-1. Microscopy

1. No
2. Yes

Q15-1a. If 'Microscopy' is covered, what is the extent of coverage for patients?

1. No coverage
2. Less than 20% of total costs covered
3. 20~50% of total costs covered
4. More than 50% of total costs covered
5. Fully covered
6. Other: _______________

Q15-1b. What is the lowest level of facility that provides this service?

1. Lower-level facility than health center (e.g., health post)
2. Health center
3. District hospital
4. Regional (provincial) hospital
5. Tertiary hospital

Q15-2. Chest X-ray

1. No
2. Yes

Q15-2a. If 'Microscopy' is covered, what is the extent of coverage for patients?

1. No coverage
2. Less than 20% of total costs covered
3. 20~50% of total costs covered
4. More than 50% of total costs covered
5. Fully covered
6. Other: _______________

Q15-2b. What is the lowest level of facility that provides this service?

1. Lower-level facility than health center (e.g., health post)
2. Health center
3. District hospital
4. Regional (provincial) hospital
5. Tertiary hospital

Q15-3. Molecular TB diagnosis (NAAT: e.g., GeneXpert)

1. No
2. Yes

Q15-3a. If 'Molecular TB diagnosis (NAAT: e.g., GeneXpert)' is covered, what is the extent of coverage for patients?

1. No coverage

2. Less than 20% of total costs covered
3. 20~50% of total costs covered
4. More than 50% of total costs covered
5. Fully covered
6. Other: _______________

Q15-3b. What is the lowest level of facility that provides this service?

1. Lower-level facility than health center (e.g., health post)
2. Health center
3. District hospital
4. Regional (provincial) hospital
5. Tertiary hospital

Q15-4. LTBI diagnosis

1. No
2. Yes

Q15-4a. If 'LTBI diagnosis' is covered, what is the extent of coverage for patients?

1. No coverage
2. Less than 20% of total costs covered
3. 20~50% of total costs covered
4. More than 50% of total costs covered
5. Fully covered
6. Other: _______________

Q15-4b. What is the lowest level of facility that provides this service?

1. Lower-level facility than health center (e.g., health post)
2. Health center
3. District hospital
4. Regional (provincial) hospital
5. Tertiary hospital

Q15-5. Molecular DST (Drug-Susceptibility Test)

1. No
2. Yes

Q15-5a. If 'Molecular DST (Drug-Susceptibility Test)' is covered, what is the extent of coverage for patients?

1. No coverage
2. Less than 20% of total costs covered
3. 20~50% of total costs covered
4. More than 50% of total costs covered
5. Fully covered
6. Other: _______________

Q15-5b. What is the lowest level of facility that provides this service?

1. Lower-level facility than health center (e.g., health post)
2. Health center
3. District hospital
4. Regional (provincial) hospital

5. Tertiary hospital

Q15-6. Phenotypic DST (Drug-Susceptibility Test)

1. No
2. Yes

Q15-6a. If 'Phenotypic DST (Drug-Susceptibility Test)' is covered, what is the extent of coverage for patients?

1. No coverage
2. Less than 20% of total costs covered
3. 20~50% of total costs covered
4. More than 50% of total costs covered
5. Fully covered
6. Other: _______________

Q15-6b. What is the lowest level of facility that provides this service?

1. Lower-level facility than health center (e.g., health post)
2. Health center
3. District hospital
4. Regional (provincial) hospital
5. Tertiary hospital

Q15-7. First-line TB treatment

1. No
2. Yes

Q15-7a. If 'First-line TB treatment' is covered, what is the extent of coverage for patients?

1. No coverage
2. Less than 20% of total costs covered
3. 20~50% of total costs covered
4. More than 50% of total costs covered
5. Fully covered
6. Other: _______________

Q15-7b. What is the lowest level of facility that provides this service?

1. Lower-level facility than health center (e.g., health post)
2. Health center
3. District hospital
4. Regional (provincial) hospital
5. Tertiary hospital

Q15-8. First-line TB treatment

1. No
2. Yes

Q15-8a. If 'First-line TB treatment' is covered, what is the extent of coverage for patients?

1. No coverage
2. Less than 20% of total costs covered

3. 20~50% of total costs covered
4. More than 50% of total costs covered
5. Fully covered
6. Other: _______________

Q15-8b. What is the lowest level of facility that provides this service?

1. Lower-level facility than health center (e.g., health post)
2. Health center
3. District hospital
4. Regional (provincial) hospital
5. Tertiary hospital

Q15-9. Preventive treatment (LTBI)

1. No
2. Yes

Q15-9a. If 'Preventive treatment (LTBI)' is covered, what is the extent of coverage for patients?

1. No coverage
2. Less than 20% of total costs covered
3. 20~50% of total costs covered
4. More than 50% of total costs covered
5. Fully covered
6. Other: _______________

Q15-9b. What is the lowest level of facility that provides this service?

1. Lower-level facility than health center (e.g., health post)
2. Health center
3. District hospital
4. Regional (provincial) hospital
5. Tertiary hospital

Q15-10. Consultation (OPD)

1. No
2. Yes

Q15-10a. If 'Consultation (OPD)' is covered, what is the extent of coverage for patients?

1. No coverage
2. Less than 20% of total costs covered
3. 20~50% of total costs covered
4. More than 50% of total costs covered
5. Fully covered
6. Other: _______________

Q15-10b. What is the lowest level of facility that provides this service?

1. Lower-level facility than health center (e.g., health post)
2. Health center
3. District hospital
4. Regional (provincial) hospital
5. Tertiary hospital

Q15-11. Hospitalization (IPD)

1. No
2. Yes

Q15-11a. If 'Hospitalization (IPD)' is covered, what is the extent of coverage for patients?

1. No coverage
2. Less than 20% of total costs covered
3. 20~50% of total costs covered
4. More than 50% of total costs covered
5. Fully covered
6. Other: _______________

Q15-11b. What is the lowest level of facility that provides this service?

1. Lower-level facility than health center (e.g., health post)
2. Health center
3. District hospital
4. Regional (provincial) hospital
5. Tertiary hospital

Q15-12. DOT

1. No
2. Yes

Q15-12a. If 'DOT' is covered, what is the extent of coverage for patients?

1. No coverage
2. Less than 20% of total costs covered
3. 20~50% of total costs covered
4. More than 50% of total costs covered
5. Fully covered
6. Other: _______________

Q15-12b. What is the lowest level of facility that provides this service?

1. Lower-level facility than health center (e.g., health post)
2. Health center
3. District hospital
4. Regional (provincial) hospital
5. Tertiary hospital

Q15-13. If there are 'Others', please specify and provide details in a short answer. (If there is nothing, please leave it blank.): _______________

Q15-13a. If 'Others (the one specified above)' is covered, what is the extent of coverage for patients?

1. No coverage
2. Less than 20% of total costs covered
3. 20~50% of total costs covered
4. More than 50% of total costs covered
5. Fully covered
6. Other: _______________

Q15-13b. What is the lowest level of facility that provides this service?

1. Lower-level facility than health center (e.g., health post)
2. Health center
3. District hospital
4. Regional (provincial) hospital
5. Tertiary hospital

Your response has been submitted. Thank you.
