# Supplementary material for: Assessment of financial protection mechanisms for tuberculosis services in seven priority countries of the Western Pacific Region: a cross-sectional mixed-methods study
Source: Trop Med Health. 2026 Aug 6;54:137. doi: 10.1186/s41182-026-00979-7 (PMC13445896; doi:10.1186/s41182-026-00979-7)
Supplement: Supplementary file 2 [file 41182_2026_979_MOESM2_ESM.pdf]

Semi-structured interview guide

No.	Question
1	Although you have already participated in the survey, could you briefly explain the financial protection policies related to tuberculosis (TB) diagnosis and treatment management?
2	Since multiple schemes (e.g., National Health Insurance (NHI) and National Health Service (NHS)) are applied in combination, how can they be distinguished in terms of eligibility for entitlements?
3	Given the mixed application of multiple schemes (NHI, NHS and Global Fund), what are the key differences from the perspective of TB patients?
4	From the patient's perspective, what are the financial challenges associated with the current financial protection system?
5	From the government's perspective, what are the financial challenges associated with sustaining the current financial protection system?
6	In the long term, if the country graduates from Global Fund support, what additional government policies should be considered?
7	In the long term, if the country graduates from Global Fund support, what would be an appropriate level of out-of-pocket expenses for TB patients?
8	Is there any communication channel available to collect feedback on the experience of using services in place (e.g., NHS services)? Does the National Tuberculosis Programme(NTP) have opportunities to regularly gather feedback from patients, especially those in rural areas, through health center staff or direct interactions with patients?
9	Please feel free to share any additional insights or important aspects that the research team may have overlooked.
